# Beamline simulations using monochromators with high *d*-spacing crystals

**DOI:** 10.1107/S160057752200707X

**Published:** 2022-08-12

**Authors:** X. J. Yu, X. Chi, T. Smulders, A. T. S. Wee, A. Rusydi, M. Sanchez del Rio, M. B. H. Breese

**Affiliations:** a Singapore Synchrotron Light Source, National University of Singapore, 5 Research Link, Singapore 117603, Singapore; b Department of Physics, National University of Singapore, Singapore 117576, Singapore; c Department of Engineering Physics, Fonty University of Applied Sciences, 5615DB Eindhoven, The Netherlands; d Centre for Advanced 2D Materials and Graphene Research Centre, National University of Singapore, Singapore 117546, Singapore; e NUS Graduate School for Integrative Sciences and Engineering, Singapore 117456, Singapore; f European Synchrotron Radiation Facility, 38000 Grenoble, France; Australian Synchrotron, Australia

**Keywords:** high *d*-spacing crystal, YB_66_, crystal monochromators, ray tracing, *SHADOW*, *OASYS*

## Abstract

Monochromators for synchrotron radiation beamlines typically use perfect crystals for the hard X-ray regime and gratings for soft X-rays. There is an intermediate range, typically 1–3 keV (tender X-rays), which common perfect crystals have difficulties covering and gratings have low efficiency, although some less common crystals with high *d*-spacing could be suitable. To evaluate the suitability of these crystals for a particular beamline, it is useful to evaluate the crystals’ performance using tools such as ray-tracing. However, simulations for double-crystal monochromators are only available for the most used crystals such as Si, Ge or diamond. Here, an upgrade of the *SHADOW* ray-tracing code and complementary tools in the *OASYS* suite are presented to simulate high *d*-spacing crystals with arbitrary, and sometimes complex, structures such as beryl, YB_66_, muscovite, *etc*. Isotropic and anisotropic temperature factors are also considered. The YB_66_ crystal with 1936 atomic sites in the unit cell is simulated, and its applicability for tender X-ray monochromators is discussed in the context of new low-emittance storage rings.

## Abstract 2

A method is introduced for representation of arbitrary crystals, numerical calculation treatment and ray-tracing simulation, and upgrading the tools in the *OASYS* suit; the upgraded tools are particularly helpful for very complicated crystals constituting charged atoms arranged in any crystalline structure and including isotropic or anisotropic temperature factors. The new open source software tools developed here are available for supporting accurate calculations in the design and optimization of new X-ray monochromators.

## 1. Introduction

X-ray monochromators use crystals that must fulfill many requirements: they must have high perfection (no dislocations, low mosaicity); be available as large, single crystals; have high resistance to radiation damage; and have high thermal conductivity. The ubiquitous material for X-ray monochromators and analyzers is silicon; crystals of silicon are available in large sizes and with high perfection. Indeed, silicon is the most perfect large crystal in the world. Germanium, with slightly higher cell parameters, also forms a highly perfect crystal but is more expensive. Synthetic diamond is also used in X-ray monochromators because of its low absorption and exceptional thermal conductivity. These are cubic face-centred-cubic (f.c.c.) crystals, and the lower non-forbidden reflection is 111, which is indeed the most used reflection in synchrotron monochromators. With a *d*-spacing of 3.135 Å, Si 111 is not very effective for large Bragg angles (3 keV corresponds to 41.2° and the minimum energy attained is 1.977 keV at normal incidence). Therefore, other crystals with large cell parameters must be investigated for applications using tender X-rays. Many natural crystals have been proposed. An exhaustive list of crystals with larger *d*-spacing is given by Underwood (2001 ▸). Databases such as *DABAX* (Sanchez del Rio, 2011 ▸ *a*) and Stepanov’s X-ray server (Stepanov, 2004 ▸) contain long lists of crystal structures that can be used in X-ray monochromators. We have compiled in Table 1 ▸ a list of crystal reflections with large *d*-spacing, including useful energy range, Darwin width (θ_d_), relative energy resolution (Δ*E*/*E*) and peak reflectivity (*R*).

Despite such a long list, it is difficult to acquire a suitable crystal for the tender X-ray regime. In particular, organic crystals cannot withstand high heat loads, and many natural crystals cannot be found with high perfection in the large size needed for monochromators. Synthetic crystals such as synthetic quartz (Cerino *et al.*, 1980 ▸; Wong *et al.*, 1999 ▸; Ohta *et al.*, 1986 ▸) and sapphire (Shvyd’ko *et al.*, 2017 ▸; Said *et al.*, 2020 ▸) are nowadays obtained with quality and size suitable for X-ray applications. However, quartz degrades very quickly under exposure to intense synchrotron radiation. Sapphire is better (Gog *et al.*, 2018 ▸), nevertheless still sensitive enough to radiation damage. Diamond has exceptional resistance that makes it appropriate for hard X-rays. The ongoing search for good crystals must be accompanied, and in many cases driven, by computer simulations of the theoretical reflectivity profiles of the crystals, to simulate by ray-tracing the whole monochromator embedded in a synchrotron beamline. Computer tools for such simulations are not easy to find, despite the already long list of available crystals in tools like *OASYS* (Rebuffi & Sanchez del Rio, 2017 ▸), or Sergei Stepanov’s X-ray server (Stepanov, 2004 ▸).

One crystal proposed and used in the tender X-ray range is YB_66_. The crystal structure is face-centered cubic with *a* = 23.440 Å (Richards & Kasper, 1969 ▸). The material is refractory and has a melting point of 2100°C. It is thermally stable and can resist severe radiation and high heat load. The crystal can be fabricated with high quality (Tanaka, 2010 ▸) and has been successfully applied in double-crystal monochromators (DCMs) in the tender energy regime (Smith *et al.*, 1998 ▸; Rek *et al.*, 1993 ▸; Wong *et al.*, 1990 ▸; Ohta *et al.*, 1986 ▸; Kitamura & Fukushima, 2004 ▸; Wong *et al.*, 1995 ▸).

About 14 years ago, at the XAFCA beamline at Singapore Synchrotron Light Source (Yu & Moser, 2008 ▸) working in a photon energy interval of 0.85–12.8 keV, we adopted a sagittal-focusing DCM with Si (111) crystals to cover the energy range 2.15–12.8 keV. However, for energies below 3 keV in the original optical design, we looked at three crystals, InSn (111), beryl ( 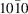 ) and YB_66_, to cover the lower-energy range. It was difficult and expensive to acquire large-size YB_66_ crystal wafers, and beryl has a strong aluminium absorption edge which is a disadvantage for the study of aluminium catalysis. A KTP (011) crystal pair was chosen in the final beamline. Ray-tracing calculations were performed using *SHADOW* (Cerrina, 1984 ▸), modified to account for crystal reflectivity interpolated from a data file, in this way inhibiting the internal crystal reflectivity calculation during ray tracing.

In recent years, larger YB-like crystals have been produced (Tanaka, 2020 ▸) at a lower cost, therefore interest in this crystal has renewed. However, no recent use of a YB_66_ crystal in a DCM beamline has been reported. One reason may be that, for high-brilliance insertion-device synchrotron sources, soft X-ray grating monochromators are now used up to 2.5 keV, close to the upper limit of tender X-rays, something that was impossible in the past (Hawthorn *et al.*, 2011 ▸; McChesney *et al.*, 2014 ▸; Tang *et al.*, 2019 ▸). In terms of the resolution and reflectivity, YB_66_ is still attractive. The YB_66_ resolving power (*E*/Δ*E*) is ∼23000 for 004 and 122000 for 006 (from Table 1 ▸), with peak reflectivity of 15% and 10%, respectively. As compared with grating monochromators, the resolution can be considered as ‘high’, and the peak reflectivity is also of the order of the efficiency obtained by good gratings. In theory, the performance obtained by a monochromator with an ideal YB_66_ crystal is at least as good as that using gratings. The use of crystals is more advantageous for large sources (wigglers or bending magnets), as gratings require a clean focusing to become effective. However, grating monochromators can easily tune the resolution by playing with the slit aperture and customizing ruling values.

To prepare for possible upgrades of synchrotron beamlines and the use of YB_66_ and other complex crystals, we have upgraded the ray-tracing code *SHADOW3* (Sanchez del Rio, 2011*b* ▸) and other tools in *OASYS* to include, in a seamless way, any crystal structure. The code algorithms and modifications are presented in this work. It is now possible with the *ShadowOUI* (Rebuffi & Sanchez del Río, 2016 ▸) add-on of *OASYS* (that interfaces *SHADOW*) to ray-trace YB_66_ crystals and any other crystal of interest for an X-ray monochromator or analyser.

## 2. Calculation of the structure factor of any crystal structure

### 2.1. Structure factor of a crystal

The structure factor of a crystal is

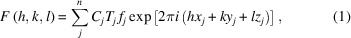

where *h*, *k*, *l* are Miller indices, *C*_*j*_ is the occupancy factor, *T*_*j*_ is the Debye–Waller or temperature factor, *f*_*j*_ = (*f*_0_ + *f*′ + *if*′′)_*j*_ are atomic scattering factors, and *x*_*j*_, *y*_*j*_, *z*_*j*_ are fractional coordinates of the atoms in the unit cell. The sub-index refers to the *j*th atom in the unit cell, and the sum extends over the *n* atoms of the unit cell.

To compute the structure factor using equation (1) for a particular structure we need libraries and methods to access:

(1) The information from the crystal structure itself, meaning a list of all atoms in the unit cell, as well as the cell parameters (*a*, *b*, *c*, α, β, γ). Each atomic center in this list must contain the fractional coordinates, the nature of the center (its atomic number *Z*) and also the ionic charge and fractional occupation. Moreover, other information could be added to compute the temperature factor, as described below. Note that the summation goes over the *n* atoms in the unit cell. In crystallography, the list of all atoms is created by applying the symmetry operations from the space symmetry group to a reduced number of atoms (the asymmetric unit).

(2) The atomic scattering factors. The elastic scattering *f*_0_ depends to a good approximation only on *q* = (sinθ)/λ, with θ the grazing incidence angle and λ the photon wavelength. The so-called ‘anomalous’ scattering factors *f*′ and *f*′′ depend on the nature of the atom and on the photon wavelength.

Although the structure factor can be efficiently calculated by a single piece of code, it requires quick access to data usually stored in libraries or databases. Four sources of information are needed: unit-cell information, *f*_0_ values, and the anomalous scattering values *f*′ and *f*′′. The *ab initio* calculation of the scattering factors can only be performed using complex quantum mechanics calculations, and it is out of the scope of most crystallography codes. Tabulated data from some references may be used, usually linking the code to available data files or databases. This linkage makes the software structure complicated and reduces portability. This is why in the ray-tracing code *SHADOW* (and also in *OASYS* crystal tools) the calculation is performed in two steps: (i) a preprocessor code that accesses necessary data from databases and creates a ‘crystal material file’ with the basic ingredients needed to build the structure factor, and (ii) the calculation of the structure factor in the *SHADOW* kernel using only the information in the preprocessor file, without any further link to databases.

The first version of *SHADOW* used internal tabulations to retrieve the *f*′ and *f*′′ values, but did not provide any *f*_0_ data. The old *SHADOW* interface in *XOP* (Sanchez del Rio, 2011 ▸ *a*) took the data from *DABAX*, an *ad hoc* compiled collection of material data, available from http://ftp.esrf.eu/pub/scisoft/DabaxFiles, where several tabulations for the same kind of data (*e.g.* *f*′) coexist. The *OASYS* package uses *xraylib* (Schoonjans *et al.*, 2011 ▸; Schoonjans, 2021 ▸) which is a compiled library that allows fast access of a large collection of X-ray data. *OASYS* can also use *DABAX* where we included the new data needed for this work.

### 2.2. Ingredients for computing the structure factor

The structure factor *F*(*h*,*k*,*l*) [equation (1)] comes from the summation of all waves scattered by the *n* atoms in the unit cell in the direction defined by the Miller indices *hkl*. Each atom *j* contributes to a wave whose amplitude is proportional to the atomic scattering factor *f*_*j*_, that measures the X-ray scattering power of each atom. Its main component is the elastic scattering factor *f*_0_. The scattered power is maximum in the direction of the incident X-rays, and decreases as a function of the angle of departure. It is proportional to the number of electrons in the atom. In ‘electron units’, *f*_0_ is equal to *Z* at the zero scattering angle (θ = 0°), and reduces to almost zero at values of *q* = (sinθ)/λ larger than about 2 Å^−1^ (2θ is the angle between incident and scattered X-ray beam with wavelength of λ). This dependency is tabulated after some theoretical calculations and can be parametrized. Cromer & Mann (1968 ▸) proposed a sum of Gaussians parametrization with nine coefficients,

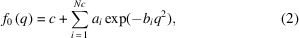

with *Nc* = 4. The nine coefficients (*a*_1_–*a*_4_, *c*, *b*_1_–*b*_4_) are obtained by fitting tabulations of *f*_0_ computed using theoretical models. A common tabulation is shown in *International Tables of Crystallography*, and includes all neutral atoms and a few ionic states. Waasmaier & Kierfel (1995 ▸) extended the number of coefficients to 11 to fit data up to *q*_max_ ≃ 6 Å^−1^. In *OASYS*, the theoretical calculation from Kissel (2000 ▸) is fitted to obtain a list of 11 coefficients for all neutral atoms. Most typical crystals used in monochromators are covalent crystals so *f*_0_ data for neutral atoms is enough. However, data for ions with integer or fractional charge is sometimes needed. For example, for YB_66_ the calculation of form factors *f*_0_ requires the ionic states ‘Y^3+^’ and ‘B^−.^’ (the dot indicates a tiny negative charge in the B atoms. We follow the formula of Higashi *et al.* (1997 ▸), so the charges removed from the neutral Y atoms are equally allocated among all boron atoms. Some *f*_0_ coefficients for ionic states are available in *International Tables for X-ray Crystallography* (Ibers & Hamilton, 1974 ▸) (hereafter called *ITC*).

For some particular ionic states, like in the case of Y^3+^ (needed in the YB_66_ crystal), the *f*_0_ data are found in the *ITC* tabulation. However, in most cases, such as for the tiny charge of B in YB_66_, there are no tabulated data for *f*_0_. Values of some ionic states of B, such as B^3+^, are found in a table in *ITC* (p. 202). We fitted B^3+^ data to obtain the nine parameters that were added to the *DABAX* file 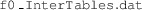 that contains the *ITC* tabulation. *DABAX* is incorporated into our tools for retrieving the nine parameters of the *f*_0_ coefficients for both neutral and ionic atoms. In cases of ions with fractional charge, like B^−0.0455^ required in the YB_66_ crystal, the nine coefficients for parametrizing *f*_0_ are calculated by interpolating the data from two entries in the table with different charges.

Using the form factors data *f*_0_(B), *f*_0_(B^3+^), *f*_0_(Y^3+^) from this reference, and assuming ‘B^−.^’ carries a negative charge of δ = 0.0455, the form factors for ‘B^−.^’ can be calculated as a linear interpolation,

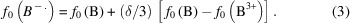

**Figure 1:**
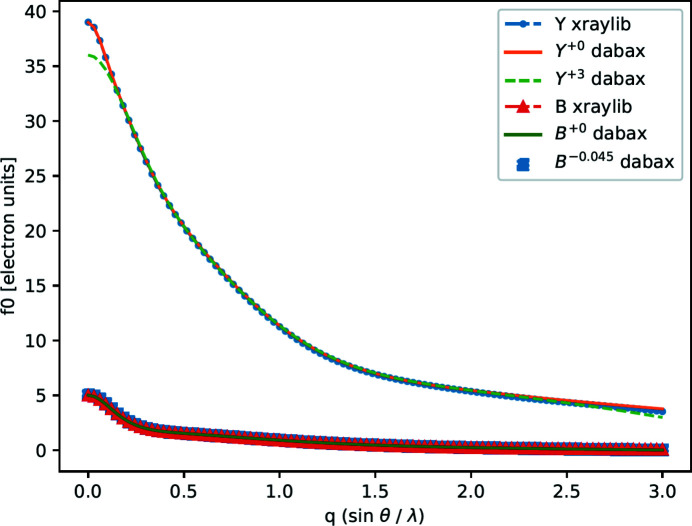
Plots of *f*_0_ values against *q* for Y and B in their neutral form (as calculated by *xraylib* and *DABAX*) and in their ionic state needed in the crystal YB_66_ (available only in *DABAX*).

A comparison plot of *f*_0_ versus *q* for neutral atoms of Y and B is shown in Fig. 1 ▸. It shows good agreement between *xraylib* and *DABAX* for the neutral species, but the new ionic states are only available from *DABAX*.

The intense elastic scattering *f*_0_ can be reduced if the incident X-ray radiation has a frequency close to the natural oscillation frequency of the electrons of a given atom. This effect is called anomalous dispersion (although there is nothing ‘anomalous’) and is represented by *f*′ and *f*′′, the real and imaginary components, respectively, of the anomalous fraction of the atomic scattering factor. These are functions of the photon energy. Anomalous scattering factors used in most software packages come from old calculations using quantum electrodynamics data (EPDL97, https://www-nds.iaea.org/epdl97/), mixed experimentally evaluated data (Henke *et al.*, 1993 ▸), or a combination of them. In the past, *SHADOW* used hybrid data from Henke *et al.* (1993 ▸) (up to 30 keV) and Cromer (1983 ▸). *DABAX* files contain many tabulations available in the literature. The library *xraylib*, used by default in *OASYS*, accesses a selected tabulation of data as described by Schoonjans *et al.* (2011 ▸). The inclusion of anomalous scattering factors in calculations of crystal diffraction profiles using the dynamical theory of diffraction is essential, as it contributes to the peak intensity.

The list of atoms in the unit cell is tabulated in the *DABAX* file 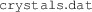 . The same data are also integrated in *xraylib*. We added the new entry YB_66_ in *DABAX*, with information on the composition of the crystal unit cell.

### 2.3. Anisotropic temperature factors

**Figure 2:**
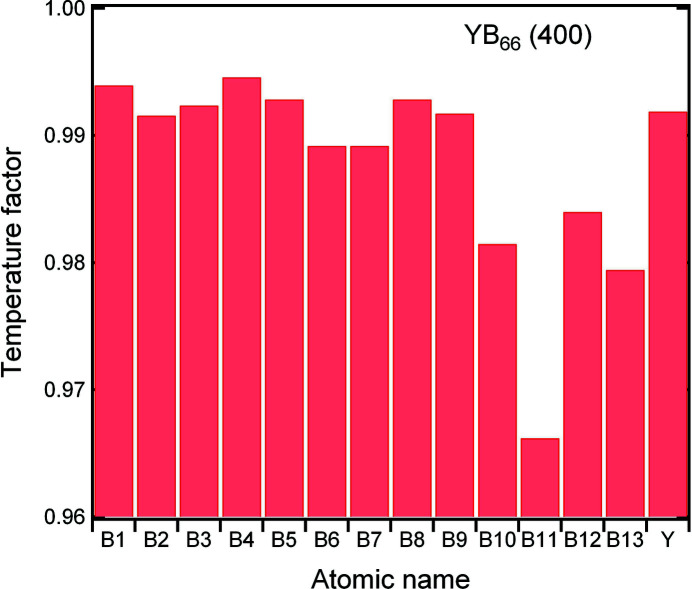
Anisotropic temperature factor of 14 symmetric sites for YB_66_ (400), each site labeled with atomic name and site index. The temperature factors vary on symmetric sites for 13 boron atoms, except B6 and B7 for which they are the same.

Displacement of atoms from their mean positions in the crystal structure weakens the scattered X-ray intensity. A Debye–Waller factor takes this effect into account in the structure analysis. Lonsdale & Grenville-Wells (1956 ▸) observed that Debye–Waller factors (also called temperature factors) for most crystals are indeed anisotropic. In addition, thermal movement from the same atoms may be different in different atomic sites (as shown in Table 2 ▸). In most cases, atoms belonging to the same group of prototypical atoms have different temperature factors. The anisotropic temperature factors of 14 symmetric sites for YB_66_ are plotted in Fig. 2 ▸; one can see that the temperature factors for boron atoms are different in 13 atomic sites, except sites B6 and B7. The anisotropic factors for all symmetric sites are necessarily included for accurate crystal structure calculations; therefore the list of prototypical atoms must also reflect the site information from the temperature factors.

To describe the anisotropic temperature factor when the atomic displacement follows a tri-variate Gaussian probability distribution function (as usually accepted), six coefficients, β_*ij*_ (*i*, *j* = 1, 2, 3), are needed [equation (21) in Trueblood *et al.* (1996 ▸)],

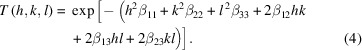

In Table 2 ▸, we give an example input of anisotropic temperature data for YB_66_ (Richards & Kasper, 1969 ▸). Boron atoms occupy 13 symmetric sites, each having different anisotropic temperature factors, and yttrium atoms occupy a single site, with equivalent temperature factors. The β_*ij*_ coefficients are always far less than 1 for an anisotropic temperature factor. Once β_11_ is set to 1, the β_*ij*_ coefficients are for a different notation, with only β_22_ representing an isotropic *B*_eq_ factor, while the β coefficients in other columns are discarded (all set to zero). The temperature-factor data for any crystal can be easily defined accordingly. If no input line beginning with 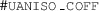 is found in the crystal file, the default scale temperature factor is used.

In the calculations, two temperature factors are implemented – the anisotropic [equation (4)] and the isotropic temperature factors. In the last case, the displacement amplitudes are equivalent for all directions, and the temperature factor depends on the isotropic *B*_eq_ factor (Trueblood *et al.*, 1996 ▸; Higashi *et al.*, 1997 ▸),

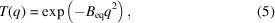

where

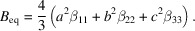

When calculating the anisotropic temperature factor for YB_66_ 400, according to the formula, only β_11_ contributes to the temperature factor, and there are two identical pairs of β_11_; therefore we only have 12 different anisotropic temperature factors. For isotropic factor *B*_eq_, there are two identical factors for sites B2 and B7, so there are 13 different temperature factors (see Fig. 2 ▸).

For some crystal structures, for instance some of the YB_66_ variants in Higashi *et al.* (1997 ▸), only *B*_eq_ factors are given. We handle such cases using the 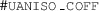 coefficients, with the convention of setting β_11_ to a number equal to or greater than one, and setting β_22_ to *B*_eq_.

### 2.4. The new crystal preprocessor for *OASYS* and *SHADOW*

The preprocessor file in *SHADOW* and *CRYSTAL* contains the elements of the structure factor [equation (1)]. The idea is to group atoms that are identical in all parameters except in (*x*, *y*, *z*) in a single ‘prototypical’ atom. With this, the number of terms in the sum is reduced and the calculation is faster.

The new code *bragg_calc2* is used for generating the crystal material file used in both *SHADOW* and *OASYS* (*CRYSTAL* and *FH* widgets). This code can use two libraries for material constants (*xraylib* and *DABAX*), at the users’ choice. When calculating YB_66_, *DABAX* is the only option, as it was upgraded with its crystal unit-cell structure (in the file 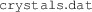 ) and *f*_0_ ionic states (in the file 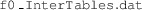 ).

**Figure 3:**
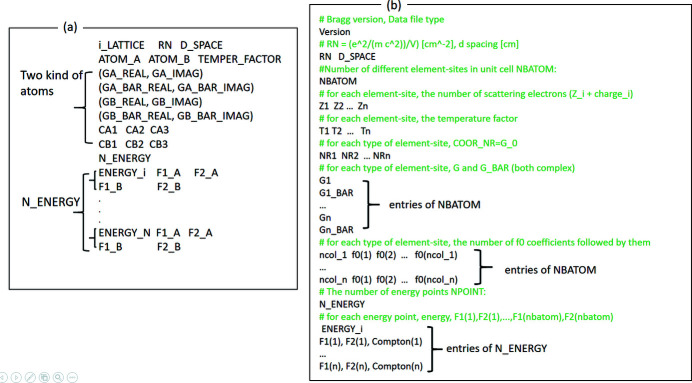
The original (*a*) and new (*b*) formats of a *SHADOW* preprocessor file. The new format file includes a comment explanation following each line of parameters. The index or the prototypical atoms goes from 1 to 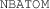 , and 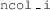 represents the number of *f*_0_ coefficients for a particular prototypical atom (it must be an odd number, typically 9 or 11).

The format of the new crystal material file evolved from the original one to minimize changes of source code; the old and new formats are shown in Figs. 3 ▸(*a*) and 3(*b*), respectively. In the old format preprocessor file, a maximum of two kinds of atoms and two symmetric sites can be input, and a maximum of eight atoms in a Bravais unit cell. The first item, named 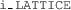 in the first line, identifies the file format. Integer values of 0 to 5 denote this file in the old format containing data for the Bravais lattice types of zinc blende, rock salt, simple f.c.c. and CsCl structure, and two hexagonal (close-packed structure and graphite structure), respectively. 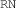 and 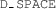 are the product of the inverse of the volume of a unit cell and classical electron radius, and the distance between crystalline planes, respectively.

In the new file format, there are three main differences for crystal input. First, there is no limit on the number of prototypical or site-atoms used; each one will have corresponding values of 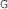 and 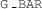 (complex conjugate of 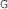 ) geometrical factors [corresponding to the exponential in equation (1)], and coefficients for *f*_0_. Second, the atoms in a crystal can carry integer or fractional charges, 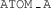 and 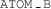 in Fig. 3 ▸(*a*). The integer atomic numbers of the constituent atoms are replaced by a float number of scattering electrons [atomic number plus ionic charge 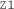 , 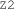 , 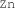 in Fig. 3 ▸(*b*)]. Third, temperature factors are included for different symmetric sites.

In the new file format, each prototypical atom has a different identifier. This contains the atomic number, site occupation, fractional charge and temperature factor. Two atoms in the unit cell belonging to the same prototype only differ in the coordinates. Therefore, equation (1) becomes a sum to *M* prototypical atoms,

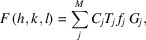

with *G* a geometrical factor that contains the sum of atoms belonging to each prototypical group,

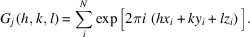

The new file format can safely replace the old file format without introducing errors for any crystal allowed in the old *SHADOW* file, because the ray-tracing calculated results are identical for the existing crystals represented by the two formats as demonstrated in Section 4.

### 2.5. New crystals in *OASYS* widgets

**Figure 4:**
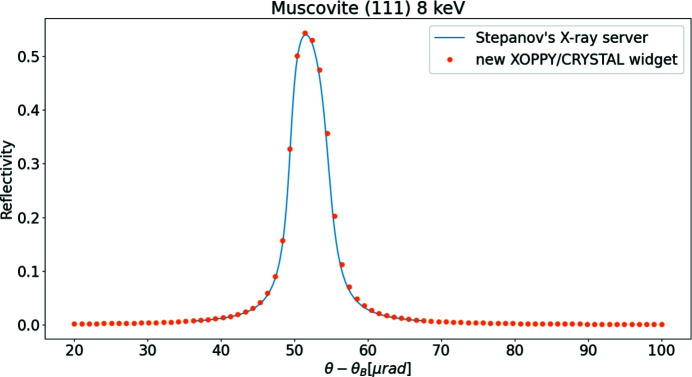
Comparison of crystal profile calculations of mica 111 at 8 keV using the new code (circles) and Stepanov’s X-ray server (solid line).

In the *OASYS* suite, the widgets that perform crystal calculations are *FH* and *CRYSTAL* in the *XOPPY* add-on, and *BRAGG* pre-processor in the *ShadowOUI* add-on. *FH* and *CRYSTAL* widgets are used for calculating crystal structure parameters, for instance, the structure factor, Darwin width, Bragg angle, *etc*. and the X-ray reflectivity for crystals. Previously these applications accepted crystals composed of neutral atoms with single scale temperature factors for all atomic sites. A new software component 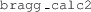 has been introduced to calculate arbitrary crystals including charged atoms, and isotropic or anisotropic temperature factors in different sites. Fig. 4 ▸ shows the reflectivity of mica 111 at 8 keV calculated with the new 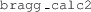 and compared with calculations using Stepanov’s X-ray server. Both curves are in good agreement.

The *SHADOW* preprocessor widget *BRAGG* is used for creating the crystal input file for *SHADOW*. This widget has been updated to use 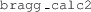 code for new crystals such as YB_66_.

## 3. Benchmarking and diffraction profiles for YB_66_ and other crystals

### 3.1. Validation of the model for the YB_66_ crystal

**Figure 5:**
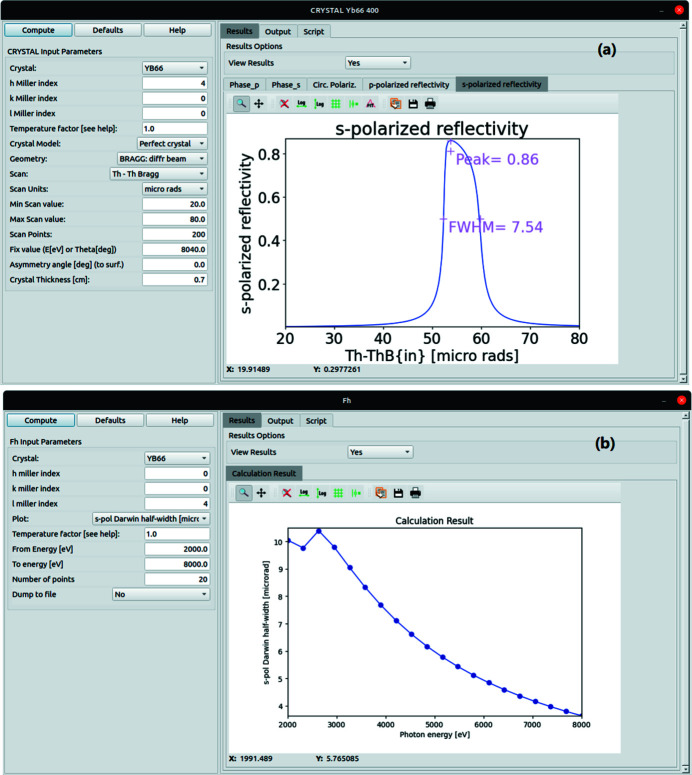
*OASYS* widgets *CRYSTAL* (*a*) and *FH* (*b*) with calculations of the reflection profile and Darwin widths for YB_66_.

To validate the generated model (lattice constants, atomic coordinators list and temperature factor, *etc*.) of YB_66_ crystal insertion in the file 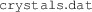 for the *OASYS* suite, the structure factors *F*(0,0,0) and *F*(*H*,*K*,*L*) are calculated with the updated *FH* widget under *XOPPY Optics* in the *OASYS* suite (see Fig. 5 ▸). Some calculated structure factors for the index planes with practical synchrotron radiation applications are tabulated in Table 3 ▸ for comparison with data from Richards & Kasper (1969 ▸), who tabulated |*F*|_CALC_ (calculated) and |*F*|_OBS_ (measured) with Cu *K*α radiation (∼8040 eV). A photon energy of 8040 eV was used in our calculations. The calculated *F*(0,0,0) of 8846 is in agreement with the calculated value of 8841 by Richard & Kaspar (1969 ▸). However, to make the overall comparison consistent, the *F*(*H*,*K*,*L*) values by Richard & Kaspar (1969 ▸) must be multiplied by four, because their calculation included the factor 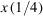 for YB_66_ (Tanaka, 2020 ▸).

### 3.2. Diffraction profiles

**Figure 6:**
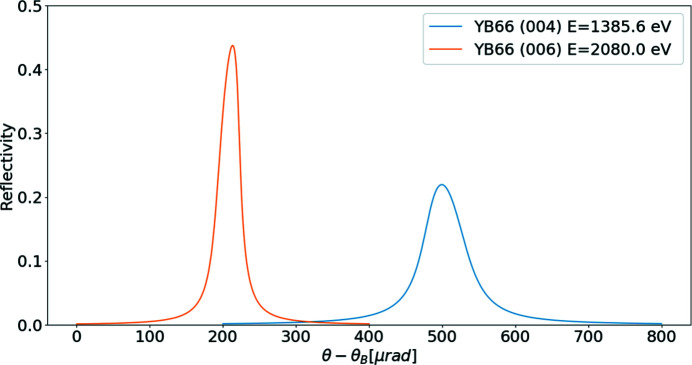
Diffraction profiles for YB_66_ 004 at 1385.8 eV and YB_66_ 006 at 2080 eV using the anisotropic temperature factor. Peak values are 0.22 and 0.44; FWHM values are 64.1 µm and 32.0 µm, respectively.

The calculated structure factors for YB_66_ are compared in Table 3 ▸ with those of Richards & Kasper (1969 ▸). The updated *CRYSTAL* widget can calculate the reflectivity for an arbitrary crystal. The reflectivity plot of YB_66_ 004 (see Fig. 6 ▸) takes into account the different anisotropic temperature factors at 14 symmetric sites and ionic atoms at 8.04 keV. No data for YB_66_ reflectivities are available in the literature for comparison. The YB_66_ 400 reflection is used in the following section in *SHADOW* ray-tracing simulation calculations, and the results are compared with the measurement data from the references.

## 4. Demonstration of ray-tracing simulations for YB_66_ crystal

The *SHADOW* code is a well known ray-tracing engine for beamline design in the synchrotron radiation community. Almost all current beamlines benefitted from the help of *SHADOW*. We use *SHADOW3* code interfaced in the *ShadowOUI* add-on of *OASYS* to demonstrate the ray-tracing results of a DCM with neutral atomic crystal and ionic atomic crystals represented by new *SHADOW* preprocessor files, in comparison with the data in the references. Both preprocessor files are created with the updated *BRAGG* widget.

**Figure 7:**
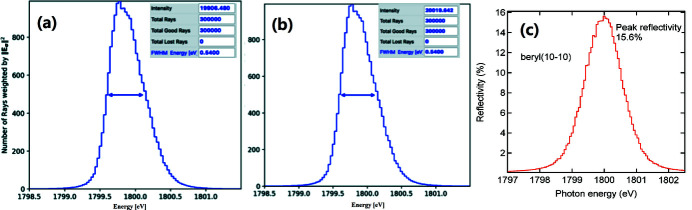
Ray-tracing plots in the *OASYS* suit for a DCM of InSb (111), using (*a*) the old and (*b*) the new format of *SHADOW* preprocessor files. (*c*) Peak reflectivity of a single beryl 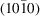 crystal which is a normalized plot between the histogram intensity on the photon energy traced after a beryl crystal and that of the source.

To check the consistency of the modifications included in *SHADOW*, we performed ray tracing of a DCM with InSb (111) crystal pair with the old and new preprocessor file formats at a photon energy around 1.8 keV. The results [Figs. 7 ▸(*a*) and 7(*b*)] show an excellent agreement in reflectance profile and full width at half-maximum (FWHM).

A second test aimed to compare simulations for beryl (Al_2_Si_6_Be_3_O_18_) with bibliographic data. Fig. 7 ▸(*c*) shows the ray-tracing result of a single-crystal reflection for beryl 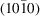 . This crystal structure includes four different elements and was not possible to simulate with the old *SHADOW*. The system comprises a point source with angular divergence of 0.3 mrad (FWHM) positioned 2 m upstream of a beryl crystal. Ray tracing gives a resolution of 0.72 eV (r.m.s.), which would correspond to a FWHM of 1.7 eV if the profile was supposed to follow a Gaussian. This is in good agreement with the measurement result of 1.6 eV FWHM [see Fig. 5(*b*) of Wong *et al.* (1999 ▸)]. Moreover, the peak reflectivity of 15.6% [see Fig. 7 ▸(*c*)] quantitatively agrees with the measurement [12.8% in Wong *et al.* (1999 ▸), Fig. 5(*a*)]. Here, the angular divergence has been intentionally set to 130 µrad RMS because this information is not found in that reference.

The last simulations concern YB_66_, and are also benchmarked against published data. The DCM of YB_66_ (004) crystals has important glitches around 1385.6 eV and 1438 eV (Smith *et al.*, 1998 ▸; Wong *et al.*, 1999 ▸). There is an increase of intensity due to the superposition of the 006 reflection at the yttrium *L*_3_ and *L*_2_ absorption edges of 2080 eV and 2156 eV, respectively. Tanaka *et al.* (1997 ▸) numerically reproduced the glitches with their custom-made program. The flux at 1385.6 eV is about 80% higher for the (004) reflection due to the added contribution of intensity at 2080 eV from the 006 reflection. We have simulated the anomalous flux enhanced at 1385.6 eV by ray-tracing calculations for the JUMBO beamline at SSRL (Cerino *et al.*, 1980 ▸) where the abnormal reflectivity was measured (Wong *et al.*, 1999 ▸). Flux measurement at JUMBO was made using a gold mesh detector (Cerino *et al.*, 1980 ▸); therefore the quantum efficiency of gold (Krumrey *et al.*, 1988 ▸) must be taken into account in the calculation. For creating the YB_66_ preprocessor files around 2080 eV it is important to use a dense energy grid. The anomalous dispersion factor (*f*′, *f*′′) must be sampled correctly, which is guaranteed using Henke data (Henke *et al.*, 1993 ▸) from the CXRO website (https://henke.lbl.gov/optical_constants/sf/y.nff), available in *DABAX*.

**Figure 8:**
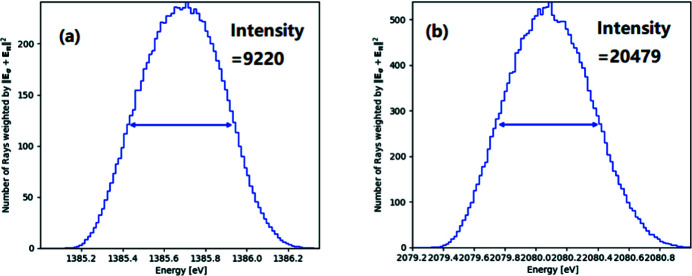
Plots of ray-tracing calculation results with five million rays for the DCM of YB_66_ (004) around 1385.6 eV (*a*) and YB_66_ (006) around 2080 eV (*b*). The input bandwidths around 1385.6 eV and 2080 eV are 0.097% and 0.086%, respectively.

For the JUMBO beamline, there was a Pt-coated mirror at an incidence angle of 89° followed by a DCM of YB_66_ (004), and a gold screen detector downstream of the DCM. The calculation parameters are listed in Table 4 ▸. The flux is calculated using parameters of the SPEAR storage ring at SSRL (Baltay *et al.*, 1991 ▸), for a beam energy of 3 GeV and bending magnetic field of 0.84 T, 0.6 mrad vertical acceptance. The flux at 1385.6 eV is normalized to unity in Table 4 ▸. The plots of ray-tracing results by the DCM of YB_66_ (004) and YB_66_ (006) at 1385.6 eV and 2080 eV, respectively, are shown in Fig. 8 ▸. The source bandwidth for source generation in the ray tracing around 1385.6 eV and 2080 eV are chosen to be 0.097% (1385–1386.35 eV) and 0.086% (2079.2–2081 eV), respectively, in order to match the DCM resolution. The obtained ray-tracing intensities are 9220 and 20479 for a source with five million rays.

The photoelectron yield intensity (*I*) created by photons incident on the gold screen detector is proportional to the product of flux (*F*), bandwidth (BW) used in ray tracing, mirror reflectivity (*R*), quantum efficiency (QE) and intensity (WI). WI is found in Fig. 8 ▸ and the actual bandwidth (BW) is normalized with the bandwidth (0.1%) used in calculation of the relative flux (*F*) in Table 4 ▸,

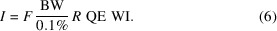

The yield ratio at the intensities at 1385.6 eV and 2080 eV can be calculated with this equation using data in Table 4 ▸ and Fig. 7 ▸, thus resulting in *I*(006)/*I*(004) = (1.01 × 0.086% × 0.59 × 0.027 × 20479) / (1.0 × 0.097% × 0.77 × 0.045 × 9220) = 0.91. This value of 91% increase in flux at 1385.6 eV quantitatively reproduces the anomalous flux glitch (80%) at 1385.6 eV for the DCM of YB_66_ (004) reported by Wong *et al.* (1999 ▸).

## 5. Summary and conclusions

The use of crystals with high *d*-spacing in synchrotron radiation monochromators is still a challenge due to the poor quality of most of the suitable crystals. However, improving technology in growing crystals makes it possible to use some of them for tender X-ray monochromators. We extend the current methods of simulating X-ray crystal reflectivity, and perform ray-tracing simulations with crystals constituting charged atoms arranged in any crystalline structure and including isotropic or anisotropic temperature factors. This will open more opportunities for numerical calculations for simulating present or future X-ray monochromators. We presented examples of X-ray reflectivity for mica, InSb and beryl. Furthermore, we analyzed in detail the YB_66_ crystal with a complex structure of 1936 atomic centers. Ray-tracing simulations were performed for a JUMBO monochromator retrieving values consistent with the experimental results. In summary, the tools available in *OASYS* for crystal reflectivity and ray-tracing crystal monochromators are upgraded to account for any crystal, once the unit cell is known. The new open source software tools developed here are available for supporting accurate calculations in the design and optimization of new X-ray monochromators.

The *OASYS* workspaces and scripts implementing the simulations presented in this paper are available at https://github.com/91902078/yb66.

## Figures and Tables

**Table 1:** Crystals with large 2*d*-spacings with the usable photon energy range, Darwin width (θ_d_), energy resolution (Δ*E*/*E*) and peak reflectivity (*R*). The lowest energy corresponds to normal incidence. θ_d_ and *R* and Δ*E*/*E* = θ_d_cot(θ_B_), where θ_B_ is the Bragg angle, are calculated at 1.1 times the lowest energy. Calculations are performed with code developed in this work scanning the crystal structures available in *DABAX* (source code 0) and Stepanov’s X-ray server (source code 1)

Source code	Crystal (*hkl*)	2*d* (Å)	Energy range (keV) (θ_d_ (µrad))	Δ*E*/*E* (×10^−3^)	*R* (%)
0	Si (111)	6.27	2.0–22.7 (259)	0.12	53
0	GaP (111)	6.29	2.0–22.6 (512)	0.23	65
1	AlP (111)	6.30	2.0–22.6 (216)	0.10	62
1	UO2 (111)	6.32	2.0–22.5 (1136)	0.52	82
1	Molybdenite (010)	6.32	2.0–22.5 (299)	0.14	70
1	Zincite (010)	6.50	1.9–21.9 (488)	0.22	48
0	NaCl (111)	6.51	1.9–21.8 (49)	0.02	26
1	Fe_3_Si (111)	6.53	1.9–21.8 (229)	0.11	12
0	GaAs (111)	6.53	1.9–21.8 (688)	0.32	57
0	Ge (111)	6.53	1.9–21.8 (691)	0.32	54
1	Co_2_FeSi (111)	6.53	1.9–21.8 (229)	0.11	11
1	FeBO_3_ (110)	6.54	1.9–21.8 (252)	0.12	58
1	AlAs (111)	6.54	1.9–21.8 (521)	0.24	65
1	ZnSe (111)	6.55	1.9–21.7 (679)	0.31	60
1	Co_2_TiSi (111)	6.63	1.9–21.5 (145)	0.07	6
1	AlFe3 (111)	6.70	1.9–21.2 (220)	0.10	12
1	Sapphire_hex (110)	6.73	1.8–21.1 (168)	0.08	38
1	CdS (111)	6.73	1.8–21.1 (681)	0.31	77
1	HgS (111)	6.76	1.8–21.1 (778)	0.36	76
0	InP (111)	6.78	1.8–21.0 (685)	0.31	79
0	CsF (111)	6.94	1.8–20.5 (590)	0.27	69
0	InAs (111)	6.97	1.8–20.4 (793)	0.36	64
1	CdSe (111)	6.99	1.8–20.4 (772)	0.35	66
1	HgSe (111)	7.03	1.8–20.2 (875)	0.40	63
0	GaSb (111)	7.04	1.8–20.2 (817)	0.37	55
1	ZnTe (111)	7.05	1.8–20.2 (820)	0.38	58
1	InN (010)	7.08	1.8–20.1 (601)	0.28	49
1	AlSb (111)	7.08	1.8–20.1 (699)	0.32	67
1	GdSb (111)	7.18	1.7–19.8 (58)	0.03	5
0	KCl (111)	7.27	1.7–19.6 (33)	0.02	17
1	LiNbO_3_ (110)	7.28	1.7–19.5 (330)	0.15	61
1	LiTaO_3_ (110)	7.29	1.7–19.5 (401)	0.18	51
1	La2CuO4_tetragonal (011)	7.37	1.7–19.3 (229)	0.11	8
1	La_0.5_Sr_1.5_MnO_4_ (011)	7.38	1.7–19.3 (58)	0.03	2
1	Sr_2_TiO_4_ (011)	7.42	1.7–19.2 (65)	0.03	7
1	Forsterite (101)	7.45	1.7–19.1 (38)	0.02	4
1	MnAs (010)	7.45	1.7–19.1 (232)	0.11	7
1	CaMnO_3_ (001)	7.46	1.7–19.1 (267)	0.12	35
1	HgTe (111)	7.46	1.7–19.1 (997)	0.46	60
0/1	InSb (111)	7.48	1.7–19.0 (898)	0.41	61
1	CdTe (111)	7.49	1.7–19.0 (891)	0.41	63
1	Cu_3_Au (001)	7.50	1.7–19.0 (442)	0.20	7
1	LaAlO_3_ (001)	7.58	1.6–18.8 (522)	0.24	30
1	LuPtBi (111)	7.59	1.6–18.8 (621)	0.28	4
1	SrVO_3_ (001)	7.68	1.6–18.5 (96)	0.04	8
1	CaRuO_3_ (001)	7.70	1.6–18.5 (497)	0.23	49
1	LaNiO_3__cubic (001)	7.71	1.6–18.4 (200)	0.09	8
1	Sr_4_Ti_3_O_10_ (011)	7.73	1.6–18.4 (41)	0.02	2
1	LaMnO_3_ (001)	7.76	1.6–18.3 (257)	0.12	15
1	LaCuO_3_ (001)	7.79	1.6–18.3 (174)	0.08	6
1	La_0.7_Sr_0.3_MnO_3_ (110)	7.79	1.6–18.3 (868)	0.40	68
1	SrTiO_3_ (001)	7.81	1.6–18.2 (62)	0.03	4
1	SrTiO_3__tetragonal (110)	7.81	1.6–18.2 (62)	0.03	4
0	YB_66_ (6 0 0)	7.81	1.6–18.2 (17)	0.01	10
1	LaFeO_3__cubic (001)	7.86	1.6–18.1 (229)	0.11	12
1	La_2_O_3__hex (010)	7.88	1.6–18.1 (526)	0.24	24
1	PbMg_0.24_Nb_0.48_Ti_0.28_O_3_r (001)	7.95	1.6–17.9 (456)	0.21	23
1	Bismuth-primitive (001)	7.98	1.6–17.8 (144)	0.07	3
1	PbMg_0.24_Nb_0.47_Ti_0.29_O_3_ (001)	8.03	1.5–17.7 (436)	0.20	26
1	LiF (001)	8.06	1.5–17.7 (162)	0.07	48
1	BaTiO_3_ (001)	8.06	1.5–17.6 (308)	0.14	23
1	Paratellurite (011)	8.13	1.5–17.5 (213)	0.10	5
1	CsCl (001)	8.25	1.5–17.3 (537)	0.25	60
1	PZT_PbZr_0.52_Ti_0.48_O_3_ (001)	8.28	1.5–17.2 (269)	0.12	38
0	LaB_6_ (001)	8.31	1.5–17.1 (509)	0.23	67
1	PbTiO_3_ (001)	8.31	1.5–17.1 (543)	0.25	30
0	α-Quartz (010)	8.51	1.5–16.7 (177)	0.08	44
1	PbMg_0.25_Nb_0.49_Ti_0.26_O_3_h (011)	8.82	1.4–16.1 (1046)	0.48	54
1	Tellurium-I (010)	8.91	1.4–16.0 (453)	0.21	12
0	Muscovite (110)	8.96	1.4–15.9 (65)	0.03	8
1	Sapphire_rhomb (011)	9.09	1.4–15.7 (190)	0.09	36
1	CsH_2_PO_4_ (001)	9.30	1.3–15.3 (187)	0.09	23
1	MgAl_2_O_4_ (111)	9.33	1.3–15.2 (254)	0.12	34
1	Li_2_B_4_O_7_ (200)	9.47	1.3–15.0 (70)	0.03	8
1	Y_3_Al_5_O_12_ (211)	9.80	1.3–14.5 (147)	0.07	8
1	Quartz (010)	9.83	1.3–14.5 (224)	0.10	38
1	PbMoO_4_ (011)	9.91	1.3–14.4 (212)	0.10	4
0	PET (011)	10.00	1.2–14.2 (140)	0.06	39
1	KDP (011)	10.17	1.2–14.0 (128)	0.06	12
1	PbZrO_3_ (110)	10.53	1.2–13.5 (344)	0.16	4
1	ADP (011)	10.66	1.2–13.3 (323)	0.15	48
1	KTiOPO_4_ (011)	10.96	1.1–13.0 (316)	0.15	19
0	KTP (011)	10.97	1.1–13.0 (316)	0.14	19
1	NaCl (001)	11.28	1.1–12.6 (206)	0.09	11
1	CaCO_3__R3c (011)	11.72	1.1–12.1 (100)	0.05	5
0	YB66 (400)	11.72	1.1–12.1 (94)	0.04	15
1	Fe_2_As (001)	11.95	1.0–11.9 (850)	0.39	3
1	KCl (001)	12.59	1.0–11.3 (189)	0.09	12
1	PbTe (001)	12.92	1.0–11.0 (456)	0.21	2
1	Bismuth-fcc (001)	13.13	0.9–10.8 (1178)	0.54	6
0	CsCl (001)	14.04	0.9–10.1 (130)	0.06	33
1	C_9_H_10_N_2_ (011)	14.36	0.9–9.9 (122)	0.06	13
0	Beryl (010)	15.74	0.8–9.0 (583)	0.27	13
0	TlAP (001)	25.76	0.5–5.5 (1091)	0.50	8
0	RbAP (001)	26.14	0.5–5.4 (954)	0.44	5
0	KAP (001)	27.70	0.4–5.1 (667)	0.31	2

**Table 2:** Anisotropic parameters for the Debye–Waller factor for the prototypical atoms of YB_66_ The atom symbols and labels are appended to the keyword 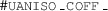 .

User parameter	Start	End	β_11_	β_22_	β_33_	β_12_	β_13_	β_23_
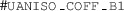	1	96	0.00038	0.00044	0.00039	0	0	0
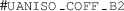	97	192	0.00053	0.00041	0.00040	0	0	0
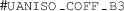	193	288	0.00048	0.00041	0.00029	0	0	0
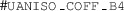	289	384	0.00034	0.00035	0.00027	0	0	0
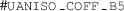	385	480	0.00045	0.00030	0.00040	0	0	0
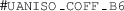	481	672	0.00068	0.00038	0.00038	0.00006	0.00009	0.00011
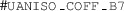	673	864	0.00068	0.00035	0.00031	−1.0×10^−5^	−6.0×10^−5^	0.00007
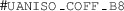	865	1056	0.00045	0.00042	0.00050	−2.0×10^−5^	0.00020	−5.0×10^−5^
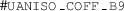	1057	1248	0.00052	0.00030	0.00076	0.00004	−1.0×10^−5^	0.00005
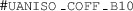	1249	1440	0.00117	0.00140	0.00131	−3.7×10^−4^	−5.1×10^−4^	−5.9×10^−4^
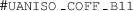	1441	1632	0.00215	0.00155	0.00359	−2.7×10^−4^	−5.6×10^−4^	0.00049
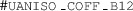	1633	1824	0.00101	0.00032	0.00111	−1.4×10^−4^	−8.4×10^−4^	0.00011
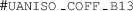	1825	1888	0.00130	0.00130	0.00130	0.00056	0.00056	0.00056
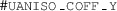	1889	1936	0.00051	0.00048	0.00048	0	0	0

**Table 3:** Comparison of calculated structures factors with those of Richards & Kasper (1969 ▸) for YB_66_ *F*(0,0,0) = 8846. |*F*| are structure factor calculations in this text. |*F*|_CALC_ and |*F*|_OBS_ are calculations and observations, respectively, taken from Richards & Kasper (1969 ▸).

*H*	*K*	*L*	|*F*|	|*F*|_CALC_	|*F*|_OBS_	|*F*|/|*F*|_CALC_
2	0	0	35.7	35.2	27.6	1.01
4	0	0	566.7	551.6	601.6	1.03
6	0	0	40.3	44.8	47.6	0.900
8	0	0	23.7	19.2	28.8	1.24

**Table 4:** Some parameters of JUMBO at the SPEAR storage ring used for calculations

Photon energy (eV)	Relative flux, *F* (0.1% bandwidth)	Mirror reflectivity, *R*	Quantum efficiency, QE
1385.6	1	0.77	0.045
2079	1.01	0.59	0.027
